# Therapeutic Potential of 2-Methylquinazolin-4(3H)-one as an Antiviral Agent against Influenza A Virus-Induced Acute Lung Injury in Mice

**DOI:** 10.3390/molecules27227857

**Published:** 2022-11-14

**Authors:** Rong Tian, Haiyan Zhu, Yan Lu, Xunlong Shi, Peng Tu, Hong Li, Hai Huang, Daofeng Chen

**Affiliations:** 1 Department of Natural Medicine, School of Pharmacy, Fudan University, No. 3728, Jin Ke Road, Shanghai 201203, China; 2 Department of Biological Medicines & Shanghai Engineering Research Center of Immunotherapeutics, School of Pharmacy, Fudan University, No. 3728, Jin Ke Road, Shanghai 201203, China; 3 Department of Pharmacy, Fudan University, No. 3728, Jin Ke Road, Shanghai 201203, China

**Keywords:** influenza A virus, 2-Methylquinazolin-4(3H)-one, antiviral activity, acute lung injury, viral pneumonia, Qingdai-Mabo

## Abstract

Qingdai-Mabo (QM), a traditional Chinese herbal formula composed of medicinal herb and fungus, has been used for treatment of cough and viral pneumonia. However, the underlying mechanism and bioactive components against anti-influenza A virus remain unclear. In the present study, ethyl acetate (EA) extract of QM decoctions was tested for its biological activity against acute lung injury (ALI) and its main components were identified using UPLC−MS/MS. In total, 18 bioactive components were identified, including 2-Methylquinaozlin-4(3H)-one (C1), which showed significant antiviral activity in vitro with an IC_50_ of 23.8 μg/mL. Furthermore, we validated the efficacy of C1 in ameliorating ALI lesions and inflammation in influenza A virus-infected mice. The results showed that C1 significantly reduced the lung index, downregulated neuraminidase (NA) and nucleoprotein (NP), and decreased the expression of pro-inflammatory molecules IFN-α, TNF-α, MCP-1, IL-6, and IL-8; however, they enhanced levels of IL-10 and IFN-γ in lung homogenate from mice infected by influenza A virus. In addition, C1 inhibited the recruitment of macrophages. These in vitro and in vivo studies suggested that the significant anti-influenza A virus activity contributed to its curative effect on lesions and inflammation of viral pneumonia in mice. Given its potential antiviral activity against influenza A virus, C1 is determined to be a main active component in the EA extract of QM. Taken together, the antiviral activity of C1 suggests its potential as an effective treatment against viral pneumonia via the inhibition of virus replication, but the mechanism C1 on antiviral research needs to be explored further.

## 1. Introduction

Influenza A virus, especially the H1N1 subtype, is a common respiratory virus that could trigger severe viral pneumonia and has great pandemic potential [1]. In addition, influenza A virus (IAV) H1N1 is a more virulent and rapidly mutating subtype with stable adaptation to humans that is responsible for seasonal epidemics each year, causing high morbidity and massive economic loss [2,3]. Focal lesions localized to the lungs were confirmed at autopsies of patients with H1N1 infection [4]. Furthermore, H1N1 infection was established to trigger excessive immune response and activate a series of robust inflammatory cascades, known as ‘cytokine storm’, in the lungs [5]. Influenza strains infecting respiratory epithelia are detected by pattern recognition receptors (PRRs). This leads to the induction of cytokine responses and the recruitment of activated immune cells by PRRs [6]. Whereas optimal levels of cytokines at the onset of viral infection can help control viral replication, sudden acute increases in cytokines triggers cytokine storm, contributing to the pathological development of viral pneumonia. In other words, the course of the H1N1 virus activates the cytokine messenger mechanism, leading to an inflammatory cascade reaction, which progresses to acute lung injury (ALI), resulting in fatal influenza disease. Clinically, ALI is characterized by hemorrhage, neutrophil infiltration, lung edema and bronchiole epithelial desquamation, progressing to a marked thickening of the alveolar wall, which eventually leads to acute respiratory distress syndrome (ARDS) [2]. In clinical practice, severe ARDS often leads to multiple organ dysfunction syndrome (MODS), which is associated with poor prognosis in ALI/ARDS patients.

Antiviral medications, such as neuraminidase inhibitors and vaccines have been successful used in clinical treatment and prophylaxis against influenza. Many antiviral drugs have been approved for the treatment of the influenza A virus, including adamantane and oseltamivir. However, owing to the rapid viral mutation rate and short therapeutic time window, the clinical application of these drugs is limited by drug resistance, undesirable toxic side effects and high cost of effective drugs [7,8]. Therefore, it is necessary to develop new safer and efficacious antiviral drug or alternative therapy for attenuating ALI symptoms in patients infected by H1N1. On this basis, efforts to discover effective agents, including immunomodulators or antivirus agents, are required to develop an adjunctive therapy for H1N1 infection.

Chinese medicine is an important medical resource for the discovery of anti-viral drugs. The formulation Qingdai-Mabo, a traditional Chinese medicine preparation composed of indigo naturalis (Qing dai) and lasiosphaera calvatia (Ma bo), was recorded in the book *Shijinmo double-herb prescriptions* as treatment for parotitis and cholic pharyngitis [9]. Moreover, our previous results showed that Qingdai-Mabo decoctions (QM) had already been proven to markedly mitigate the severity of viral pneumonia, which was attributed to its anti-complement, anti-oxidant and anti-inflammatory activity [10]. However, the therapeutic potential of QM against H1N1-induced ALI has not been elucidated yet. In this study, we applied recognition techniques (LC-MS/MS) to identify bioactive compounds in ethyl acetate (EA) extract of QM. Briefly, we found that EA extract (2.5, 5, 10 mg/kg) significantly reduced the lung index in a dose-dependent manner and inhibited the overproduction of inflammatory cytokines in H1N1-infected mice (*p* < 0.05). In addition, 18 chemical constituents were identified, some of which possessed antivirus properties. Preliminary research showed that 2-Methylquinaozlin-4(3H)-one (C1) had the strongest anti-viral property among these compounds. We also investigated the potential antiviral effect of C1 in the EA extract on influenza A replication and cytokine levels in vivo. The results suggested that C1 may be involved in attenuating the pathogenesis process of H1N1-induced ALI by directly inhibiting viral replication. Therefore, C1 isolated from QM is a major bioactive component with anti-viral activity and therapeutic potential against IAV-induced ALI.

## 2. Materials and Methods

### 2.1. Reagents

Indigo naturalis and lasiosphaera calvatia were purchased from Cambridge Yinpian Co., Ltd. (Shanghai, China). The materials were identified by the authors. EA extract of indigo naturalis and lasiosphaera calvatia aqueous were prepared in our laboratory (Department of Natural Medicine, School of Pharmacy, Fudan University), as earlier described.

The human lung cancer A549 cell line was purchased from American Type Culture Collection (Manassas, VA, USA). RPMI-1640 medium was supplied by Sigma (Shanghai, China). Ribavirin and Oseltamivir phosphate were purchased from Meryer Co., Ltd. (Shanghai, China) and Aladdin Co., Ltd. (Shanghai, China), respectively. HRP anti-influenza A virus nucleoprotein (NP) and goat anti-mouse IgG were obtained from Abcam Co., Ltd. (Shanghai, China). Neuraminidase (NA) assay kit was obtained from Beyotime Institute of Biotechnology (Jiangsu, China). A 3,3′-N-diaminobenzidine (DAB) tetrahydrochloride kit was obtained from Abcam Co., Ltd. (Shanghai, China). BCA Kit was purchased from Beyotime Biotechnology (Shanghai, China). ELISA kits for mouse interleukin-6 (IL-6), interleukin-1β (IL-1β), tumor necrosis factor-α (TNF-α), monocyte chemoattractant protein-1 (MCP-1), interleukin-10 (IL-10), interferon-α (IFN-α) and interferon-γ (IFN-γ) were purchased from Boatman Biotechnology Co., Ltd. (Shanghai, China).

### 2.2. Preparation of EA Extract

Indigo naturalis (29 kg) and lasiosphaera calvatia (11 kg) were decocted in 400 L water in triplicates for 3 h at 78 °C. The decoctions were then evaporated and dried by rotary evaporation at 50 °C in vacuum. Subsequently, the water fraction (5000 g) was collected and extracted with ethyl acetate. Finally, about 50 g extract (5%) was obtained for further research. C1 (2-Methylquinazolin-4(3H)-one), C2 (loliolide), C3 (3-(2′-Hydroxyphenyl)-2-methyl-4(3H)-quinazolinone) and C4 (clitocybin A) were isolated from EA extract using the silica gel ODS (octadecylsilyl).

### 2.3. Qualitative Analysis of Chemical Components in EA Extract

To identify active components in EA extract, chromatographic separation was performed on C_18_ column (150 × 2.1 mm, 1.9 μm, YMC, Shanghai, Japan) and their analysis was conducted using an UPLC system (Thermo, Waltham, MA, USA). The mobile phases comprised water and acetonitrile. The separation program was as follows: an isocratic elution of 2% acetonitrile for column balance for 10 min, followed by stepwise linear gradient elutions of 2–40%, 40–60%, 60–80% and 80–100% of acetonitrile from 0–40 min, 40–45 min, 45–55 min and 55–60 min, respectively. The flow rate was maintained at 0.3 mL/min, and a sample volume of 5 μL was injected. DAD (Agilent, Santa Clara, CA, USA) spectra was acquired at 190–400 nm. The device parameters were optimized as follows: positive ion mode, mass range *m*/*z* of 100–1000 Da, capillary temperature at 320 °C, capillary voltage, 30 V, sheath gas (N_2_) at 15 L/min and auxiliary gas (N_2_) at 10 L/min. Samples were diluted to a final concentration of 0.5 mg/mL with methanol. The spectra data are displayed in the Supplementary Materials (Table S1).

### 2.4. Animals and Virus

Male BALB/c mice, aged 4–6 weeks and weighing 14–16 g, were obtained from Slaccas-Shanghai Lab Animal Ltd. (Shanghai, China) (SPF II Certificate; No. SCXK 2012-0002). They were acclimated in individual ventilated cages in biosafety level II facility for one day before the experiments. All animal procedures were performed in accordance with the Animal Ethical Committee of School of Pharmacy, Fudan University (approved identification: 2016-11-SY-CDF-1). All possible efforts were taken to minimize animal suffering.

Mouse-adapted strain of high-pathogenicity influenza virus H1N1 (A/FM/1/47) was gracefully provided by associate professor Haiyan Zhu from the Department of Biological Medicines, School of Pharmacy, Fudan University. The 50% lethal dose (LD_50_) of virus in mice was tested using the Reed–Muench method (LD_50_ = 10^−5.56^ dilution of storage solution). TCID_50_ of virus was determined in cells infected with serial dilutions of the virus. The H1N1 virus was stored in aliquots at −80 °C, according to the standard procedures and protocols in our laboratory (Department of Natural Medicine, School of Pharmacy, Fudan University).

### 2.5. Determination of Antiviral Activity In Vitro

A549 human lung epithelial cell line cells were obtained from Fudan University and maintained in RPMI-1640 medium with 10% heat-inactivated fetal bovine serum (FBS) in 5% CO_2_ at 37 °C. Briefly, cells were cultured in 96-well culture plates at a density of 5 × 10^3^ cells/well. Cells were then incubated at 37 °C in a humidified atmosphere with 5% CO_2_ for 24 h. For the screening assay, A549 cells were seeded into 96-well plates to a monolayer, and then C1 diluted with DMSO to final concentrations of 0, 6.26, 12.5, 25, 50, 100, 200, 400 μg/mL were added into the wells. Afterwards, 10 μL MTT was added to each incubator/cell medium and incubated for 4 h. Culture supernatants were then collected (1000 rpm, 5 min) by centrifugation, dissolved in 100 μL DMSO and incubated for 4 h to measure at OD_490 nm_. After incubation, TC_50_ was determined for all C1 concentrations. All the MTT assays were repeated three times.

To evaluate the antiviral effect of the four QM constituents in vitro, a dose–response test of these compounds against H1N1 on Madin-Darby canine kidney (MDCK) cells was conducted. MDCK cells were cultured, as previously described [11]. Briefly, MDCK cells were cultured with DMEM supplemented with 10% fetal bovine serum (Hyclone, Logan, UT, USA), 100 U of penicillin per mL and 100 μg of streptomycin per mL. A monolayer of MDCK cells was infected with H1N1 virus (50TCID_50_) for 2 h, which was the time the cells took to fill 90% of the bottom. Subsequently, these cells were exposed to drugs at six different concentrations (200, 100, 50, 25, 12.5, 0 μg/mL). Moreover, cytopathic effect (CPE) was observed using an inverted microscope and recorded daily for three days. Half maximal inhibitory concentration (IC_50_) of the antiviral agent, which is expressed as the concentration that inhibits 50% CPE in the cultures [12], based on a comparison of the degree of the virus-induced A459 at a given concentration of antiviral agent, was calculated using GraphPad prism 6 software [13,14].

### 2.6. In Vivo Experiments of C1 on ALI Induced by H1N1 Influenza Virus

Five-week-old healthy male BALB/c mice (*n* = 6 per group) were maintained in pathogen-free individual cages with free access to drinking water and sterilized food, as described previously. The mice were randomly allocated into the following groups: normal control group, H1N1 model group, C1 (2.5, 5, 10 mg/kg) treatment group, and positive control group treated with oseltamivir (20 mg/kg, positive control). Except for normal controls, the rest of mice were intranasally infected with 30 μL of diluted H1N1virus (3LD_50_) after light anesthesia. Negative control mice (normal group) were challenged by inhaling equal volumes (30 μL) of virus diluent as the other mice. Then, 2 h later, C1 (2.5, 5, 10 mg/kg) and Oseltamivir (20 mg/kg) dissolved in 5% CMC-Na solution were given to mice (0.1 mL/10 g) once per day (i.g.) at day 0 (D0), day 1 (D1), day 2 (D2) and day 3 (D3) after infection with H1N1 virus. At the same time, normal and model groups were administered equivalent amounts of 0.5% CMC-Na in the same way. Post-infection on day 4, the mice were sacrificed to collect blood plasma, lung and liver, which were weighed. The superior right lobe of lung tissues were fixed in 10% Formalin Neutral Fixative Manual for at least 24 h to dehydrate them for subsequent histopathology analysis, whereas the rest was frozen at −80 °C for cytokine analysis [15,16].

#### 2.6.1. Organ Index Ratio Measurement and Lung Histopathology Analysis

To investigate the potential toxicity of C1 on liver and spleen and the protective effect of C1 on the lung injury in influenza virus-infected mice, mice were killed on day 4; their liver, spleen and kidney were harvested, and their liver and spleen were weighed. Organ index was calculated as follows: organ index = organ weight (mg)/body weight (g) × 100%. Lung index ratio was used to evaluate the degree of pulmonary edema.

The superior lobe of right lung was paraffin-embedded, cut into 5 μm thick sections, and stained with hematoxylin and eosin. Histopathological changes were observed under a microscope, such as cell infiltration, bronchial epithelial, thickness of alveolar walls, capillary congestion, aggregation or infiltration of inflammatory cells in airspaces and bronchial epithelial cell shedding. These were used to assess the severity of ALI and protective effect of the active component.

#### 2.6.2. Neuraminidase Activity Assay

Viral titers were measured using neuraminidase (NA) assay kit designed to detect antiviral effects. The experiment was conducted in a 96-well assay plate, following manufacturer’s protocol. A reaction mixture consisted of 10 μL lung homogenates, 70 μL detection buffer, 10 μL Mill-Q water and 10 μL NA fluorescence substrate in a 96-well plate. Briefly, after vibration mixing for approximately 1 min, the mixture was incubated at 37 °C for 30 min. The fluorescence of the mixture was quantified at 322 nm of excitation wavelength and 450 nm of emission wavelength with a muti-mode microplate read (Waltham, VT, USA). The NA activity experiment was conducted in triplicate. Oseltamivir phosphate was used as a positive control.

#### 2.6.3. Ionized Calcium Binding Adaptor Molecule-1 Assay Using Immunohistochemistry

Influenza virus load, expression of NPs and ionized calcium binding adaptor molecule-1(Iba-1) were determined using IHC. Lung sections were first deparaffinized and rehydrated with a classed series of xylene and graded alcohols. Then, the tissue sections were subjected to antigen retrieval through immersion in 10 mM sodium citrate. After quenching endogenous peroxidase activity with 3% hydrogen peroxide in methanol for 20 min in the dark, sections were blocked with 5% BSA for 1 h. Then, slides were incubated with mouse antibody (1:500, Abcam, Shanghai, China) at 4 °C overnight and, subsequently, with HRP-conjugated goat anti-mouse IgG antibody for 30 min. Finally, sections treated with chromogenic substrate solution diaminobenzidine (DAB) and counter-stained with hematoxylin were visualized under the microscope (CX41RF; Olympus, Tokyo, Japan).

#### 2.6.4. Measurement of Cytokines in Lung Tissue Homogenates

Pulmonary tissue homogenates were collected from mice lungs by centrifugation at 4500 rpm for 20 min. BCA assay was used to quantify the total protein in the supernatant of lung homogenates. Cytokine levels of IL-1β, IL-6, TNF-α, IL-10, MCP-1, INF-γ and INF-α were determined, as instructed by the manufacturer.

### 2.7. Statistical Analysis

All statistical analyses were performed using SPSS 17.0 software. Data were expressed as the mean ± standard, and differences among variables were evaluated using one-way ANOVA followed by Dunnett’s test. A value of *p* ≤ 0.05 was considered statistically significant.

## 3. Results

### 3.1. Preparation and Physicochemical Characterization of C1

**Figure 1:**
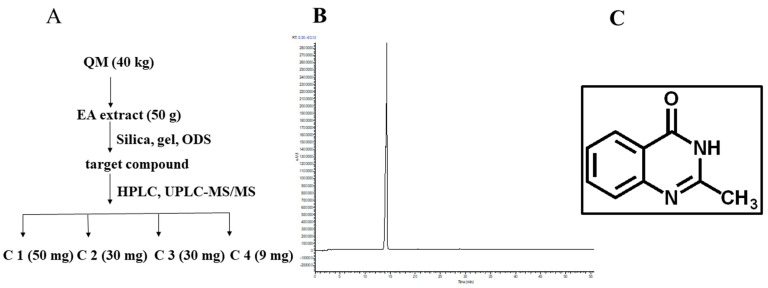
Preparation C1 in QM. (**A**) Enrichment of target compound from QM decoction; (**B**) HPLC chromatogram of C1; (**C**) Structure of compound 1. C1 was isolated using silica, gel and ODS, and further tracked by UPLC−LTQ−MS and purified preparative HPLC. A white amorphous power, with a molecular of C_9_H_8_N_2_O, which was determined by the ESIMS ion at *m*/*z* 161.16. The UPLC−MS^n^ fragments (160.99, 144.94, 120.08) indicated that C1 is a quinazolinone alkaloid.

We successfully obtained a stable EA extract of QM decoction. Moreover, 18 chemical components of QM were identified using UPLC-ESI-LTQ-MS. Based on the characteristics of chemical structure, these compounds were divided into four groups: alkaloids (1, 2, 4, 5, 6, 10–18), nucleosides (9), monoterpenes (3, 7) and sterols (8); details were attached in Supplementary Materials. Four compounds, including C1, were isolated from the EA extract using silica, gel, ODS and HPLC, as shown in Figure 1A. By comparing the retention times of the HPLC peak of the C1 sample and the standard, the four major compounds in QM decoctions were identified as C1 (2-Methylquinazolin-4(3H)-one), C2 (loliolide), C3 (3-(2′-Hydroxyphenyl)-2-methyl-4(3H)-quinazolinone) and C4 (clitocybin A).

C1 was a white power soluble in sterile water. Quantification using the UPLC-MS/MS method showed that the total C1 content in the EA extract was 1.41%. In addition, evaluation of characteristic ion fragments of C1 indicated that its molecular weight and molecular formula were 160 (Table S1) and C_9_H_8_N_2_O, respectively. NMR data were as follows: ^1^H-NMR (CDCl_3_, 400 MHz): δ_H_ 8.17 (1H, d, *J* = 8.0 Hz, H-5), 7.80 (1H, dd, *J* = 8.4, 7.0 Hz, H-7), 7.60 (1H, d, *J* = 8.2 Hz, H-8), 7.49 (1H, dd, *J* = 8.0, 7.0 Hz, H-6), 2.45 (3H, brs, H-9). The ^13^C-NMR (CDCl_3_, 100MHz): δ_C_ 162.9 (C-4), 155.0 (C-2), 148.4 (C-8a), 134.5 (C-7), 126.2 (C-5), 125.7 (C-6, C-8), 120.2 (C-4a), 20.0 (C-9).

C2 was a white power. ESI: [M+H]^+^ at *m*/*z* 197; NMR data were as follows: ^1^H-NMR (DMSO-d_6_, 400 MHz): δ_H_ 1.20 (3H, s, H-9), 1.22 (3H, s, H-10), 1.52 (3H, s, H-11), 2.35 (1H, brs, H-4), 1.27 (1H, t, *J* = 11.5 Hz, H-4), 1.15 (1H, t, *J* = 12.5 Hz, H-2), 1.87 (1H, brd, *J* = 14.2 Hz, H-2), 3.98 (1H, m, H-3), 5.82 (1H, s, H-7). The ^13^C-NMR (DMSO-d_6_, 100MHz): δ_C_ 182.2 (C-6), 171.4 (C-8), 112.8 (C-7), 86.9 (C-5), 63.4 (C-3), 50.1 (C-2), 48.3 (C-4), 35.2 (C-1), 30.2 (C-9), 25.7 (C-11), 25.1 (C-10).

C3 was a yellow power. ESI: [M+H]^+^ at *m*/*z* 253, NMR data were as follows: ^1^H-NMR (DMSO-d_6_, 400 MHz): δ_H_ 8.10 (1H, d, *J* = 7.9 Hz, H-5), 7.83 (1H, dd, *J* = 7.3, 8.0 Hz, H-6a), 7.65 (1H, d, *J* = 8.1 Hz, H-7), 7.51 (1H, dd, *J* = 7.0, 8.0 Hz, H-6), 7.31 (1H, dd, *J* = 7.8, 7.0 Hz, H-4′), 7.26 (1H, d, *J* = 7.8 Hz, H-3′), 7.06 (1H, d, *J* = 8.2 Hz, H-6′), 6.93 (1H, dd, *J* = 8.3, 7.0 Hz, H-5′). The ^13^C-NMR (DMSO-d_6_, 100MHz): δ_C_ 161.4 (C-4), 155.6 (C-2), 153.3 (C-2′), 147.9 (C-8a), 134.9 (C-7), 130.8 (C-4′), 130.0 (C-3′), 127.1 (C-8), 126.8 (C-6), 126.8 (C-5), 125.2 (C-1′), 121.1 (C-4a), 120.0 (C-5′), 117.2 (C-6′), 23.6 (C-9).

C4 was a white power. ESI: [M+H]^+^ at *m*/*z* 258, NMR data were as follows: ^1^H-NMR (CD_3_OD 600 MHz): δ_H_ 4.71 (2H, s, H-3), 6.51 (1H, d, *J* = 2.0 Hz, H-5), 6.71 (1H, d, *J* = 1.94 Hz, H-7), 6.83 (2H, d, *J* = 8.87 Hz, H-10, 12), 7.53 (2H, d, *J* = 8.81 Hz, H-10, 12). The ^13^C-NMR (CD_3_OD, 150MHz): δ_C_ 169.89 (C-1), 160.69 (C-6), 156.43 (C-4), 154.58 (C-11), 135.97 (C-8), 132.40 (C-7a), 124.25 (C-9, 13), 120.00 (C-3a), 116.57 (C-10, 12), 107.44 (C-7), 101.63 (C-5), 50.86 (C-3).

### 3.2. Antiviral Activity of C1 from EA Extract against IAV H1N1 In Vitro

**Figure 2:**
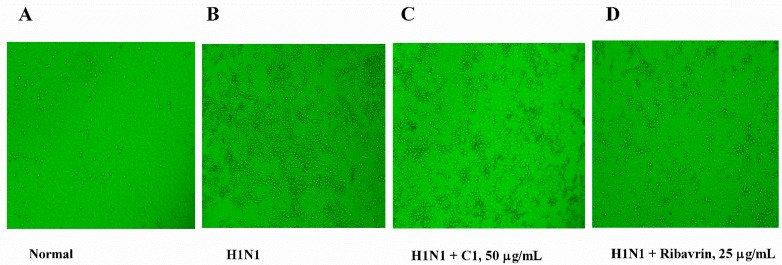
The antiviral activity of C1 isolated from EA extract against the cytopathic effect of the H1N1 virus. A monolayer of MDCK cells was infected with the H1N1 virus (50 × TCID50) by the time the cells fulfilled 90% of the bottom. After 2 h of incubation, the supernatant was carefully removed, and a sample diluted by 1640 medium was added to each well. Virus-induced CPEs are recorded each day until all the cytopathic cultures are in virus control. Inhibition assay of cytopathic effects (CPE) was evaluated to ascertain the antiviral activity of C1 and tissue culture infective dose (TC_50_), and the 50% effective concentration (IC_50_) was used to measure by regression analysis. (**A**) MDCK control; (**B**) H1N1 control; (**C**) H1N1 + C1; (**D**) H1N1 + ribavirin; Magnification: 200×.

Some of the compounds (C1–C4) isolated were used to evaluate the antiviral activity of EA extract in MDCK cell lines infected by H1N1 virus. Specifically, four compounds were tested for antiviral activity against IAV H1N1 in MDCK cells (Figure 2). CPE was indirectly used to monitor the ability of compounds to inhibit viral replication and infection. The results revealed apoptosis or shrinkage in the majority of infected cells, with parts of cells detaching to form the culture dish. In contrast, compounds with antiviral activity protected host cells from the CPE of the virus. C1 and C3 had a TC_50_ of 79.7 μg/mL and 200 μg/mL, respectively, and both compounds exhibited a direct antiviral effect in vitro, with a 72 h IC_50_ of 23.8 μg/mL and 100 μg/mL on H1N1, respectively. The main data for anti-virus activity are listed in Table 1. However, C2 and C4 did not exert any antiviral activity.

### 3.3. C1 Alleviates IAV-Induced Acute Lung Injury

**Figure 3:**
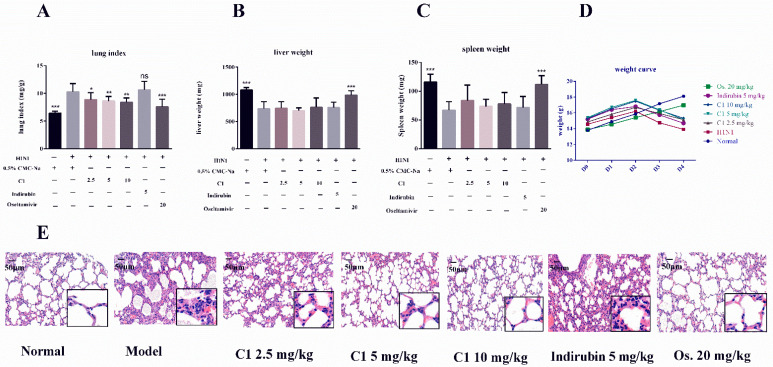
The protective effects of C1 on lung injury, lung index, organ weight and body weight. Mice were infected with 3L_50_ of the H1N1 virus and administered orally with C1, indirubin, oseltamivir or 0.5% CMC-Na at indicated doses once daily for four days. Lung index, liver weight, spleen weight and mice body weight were detected and calculated. Hematoxylin-eosin stain (H&E) with different visions (**A**) lung index = lung weight (mg) body weight (g) × 100%, (**B**) weight of liver, (**C**) weight of spleen, (**D**) body weight change curve (*n* = 42), (**E**) H&E staining, photographed at 200× and 400× magnification. Data are expressed as mean ± S.D. * *p* < 0.05, ** *p* < 0.01, *** *p* < 0.001, compared with model group.

C1 isolated from EA extract was identified as an alkaloid compound with significant antiviral properties. Here, the protective effect of C1 was investigated in mice infected with the H1N1 virus. As shown in Figure 3A, the protective effect of C1 on ALI was validated in H1N1-infected BALB/c mice. After influenza A virus attacks, the lung index in the H1N1 virus (10.27 ± 0.50 mg/g) was significantly higher, compared with the normal group (6.40 ± 0.14 mg/g, *p* < 0.001). This result suggested that IAV contributed to the severity of lung lesions. The higher lung index was significantly attenuated in the treatment group that received three C1 dosages (2.5, 5, 10 mg/kg), compared with the model control (lung indexes: 8.85 ± 0.47 mg/g, 8.60 ± 0.29 mg/g, 8.36 ± 0.28 mg/g, respectively, *p* < 0.001), especially the 10 mg/kg group. These data suggested that C1 from the EA extract could suppress severe lung edema and injury induced by H1N1, which showed a dose-dependent relationship.

Immunological system activation possibly contributes to lung tissue damage in severe viral infection. The examination of the lung section indicated that the normal group of mice had normal lungs in terms of nature, color, size and texture with intact alveolar cells (Figure 3E). Comparatively, we observed thickened alveolar wall, with inflammatory cells infiltration in lung interstitium in the model group (Figure 3E). These histological damages were significantly less in the lungs of mice receiving C1 and Os. (Figure 3E). These data indicated that the administration of C1 could ameliorate ALI induced by H1N1 virus infection. As shown in Figure 3E, no evident histological alteration was observed in lung specimens of the indirubin group (5 mg/kg), compared with the model group.

### 3.4. Protective Effects of C1 on Liver and Spleen in Influenza A Virus-Infected Mice

Pandemic influenza H1N1 strain caused multi-organ damage, interfering with several biological processes [17]. Weights of liver and spleen of mice in the model group (liver weight: 738.50 ± 40.17 mg, spleen weight: 66.78 ± 4.94 mg) were significantly lower compared with those of the normal group (liver weight: 1080 ± 18.43 mg, spleen weight: 115.80 ± 5.51 mg; *p* < 0.05), which suggested that influenza A virus could induce severe injury in vital organs. Notably, the oral administration of C1 could effectively reverse those changes. As shown in Figure 3B,C, a high dosage of C1 and Os. increased liver weight from 760.80 ± 61.88 to 986.50 ± 32.99 mg and spleen weight from 77.71 ± 7.57 to 111.50 ± 6.37 mg. Comparatively, the organ weights in the virus-infected group were lower (liver weight: 738.50 ± 40.17 mg; spleen weight: 66.78 ± 4.94 mg), although the difference was not statistically significant. Higher liver and spleen weights after treatment with the C1 suggested that the compound was therapeutically beneficial to mice infected by H1N1 virus and had no toxicity on these organs. A five-day change in body weight mice is shown in Figure 3D, the weight rate of mice raised 31.2%, 2%, 0.2% and 21.6% in normal, C1 2.5 mg/kg, C110 mg/kg and Os. 20 mg/kg groups between days 0–4, meanwhile, the weight rate of mice decreased 2% and 4.5% in the C1 5 mg/kg and model group. Treatment with C1 2.5, 10 mg/kg and Os. 20 mg/kg effectively prevented weight loss and produced a slight weight on day 4, compared with baseline weight.

### 3.5. C1 Inhibits IAV Replication in Mice

Viral virulence and host defense factors, such as type Ι interferon, determine the outcome of infected mice. To assess the effects of C1 on viral replication, we calculated the viral load based on the activity of NA and the expression of nucleoprotein (NP). Furthermore, NA, a surface glycoprotein in the influenza virus, has been demonstrated to hydrolyze the sialic acid, facilitating the release of newly assembled viruses into the host cell [18].

**Figure 4:**
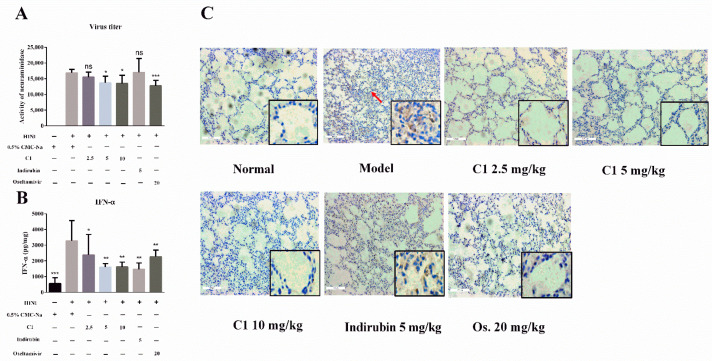
Suppression of H1N1 viral replication in vivo by C1, the experimental protocol is the same as Figure 3. (**A**) The NA activity in lung homogenates by fluorescence effect; (**B**) The expression level of INF-α in lung homogenates by ELISA; (**C**) The expression of NP in lung tissue, the representative IHC pathological images of the lung on day 4 post-infection, the red arrow indicates viral N protein-positive signal. Magnification: 200× and 400×; scale bars, 100 μm. * *p* < 0.05, ** *p* < 0.01, *** *p* < 0.001, compared with model group.

In our experiment, NA activity was used to indicate the viral load of host cells and the replication of the influenza virus [19,20]. As shown in Figure 4A, NA activity was markedly higher in the model group than in the normal control and significantly lower in the C1 group (5, 10 mg/kg) than in the model group (*p* < 0.05). However, no differences were observed between the NA activity (2.5 mg/kg) group and the model control. These data suggested that C1 at dosages of 5–10 mg/kg could inhibit the replication of the influenza virus in mice.

Viral NP has been suggested as a candidate factor regulating the switch from transcription to replication. In addition, NP interacts directly with many host cell factors and has inhibitory effects on a large host range [21,22]. As is shown in Figure 4C, instillation with H1N1 resulted in significant lung injury, whereas no apparent changes in lung pathology and NP protein deposition were observed in mice in the normal group. In addition, many viral N protein-positive signals were found in alveolar cells and lymphocytes and inflammatory cells in H1N1 virally infected mice (solid arrows), notably, viral replication ability increased after the introduction of NP. In contrast, fewer positive signals were observed in the lung tissues of C1-treated mice, compared with lungs from indirubin-treated mice and the model group. These results suggested that C1 treatment could reduce viral titers in H1N1 virus in the lungs of infected BABL/c mice.

During viral infection, type Ι interferon is up-regulated to mediate immune response and effectively inhibit the replication of IAV [23]. IFN-α, a type Ι interferon, plays a vital role in restricting the replication and spread of IAV infection. As part of the host immune system, IFN-α can recognize and inhibit viral components via the extracytosolic or cytosolic pathway. The multitude of IFN-α molecules may reflect a need to protect the host against a large array of viruses. IAV infection triggers elevated cellular immune responses mediated by the up-regulation of major histocompatibility complex (MHC) class Ι and II antigens by type Ι interferons, contributing to the overall antivirus response. The present study has demonstrated that the production of IFN-α was remarkably decreased after treatment with C1. This result was consistent with our preliminary research; indigo naturalis decoction administration significantly reduced IFN-α expression when compared with H1N1 group (*p* < 0.05) [9]. As shown in Figure 4B, the level of responsive cytokine was significantly lower in the infected mice that did not receive C1, compared with the infected mice administrated with C1. With these data, we predicted that C1 confers a protective effect on lung injury due to its anti-virus activity.

### 3.6. Effect of C1 on Cytokine Production and Recruitment of Macrophage in IAV-Infected Mice

**Figure 5:**
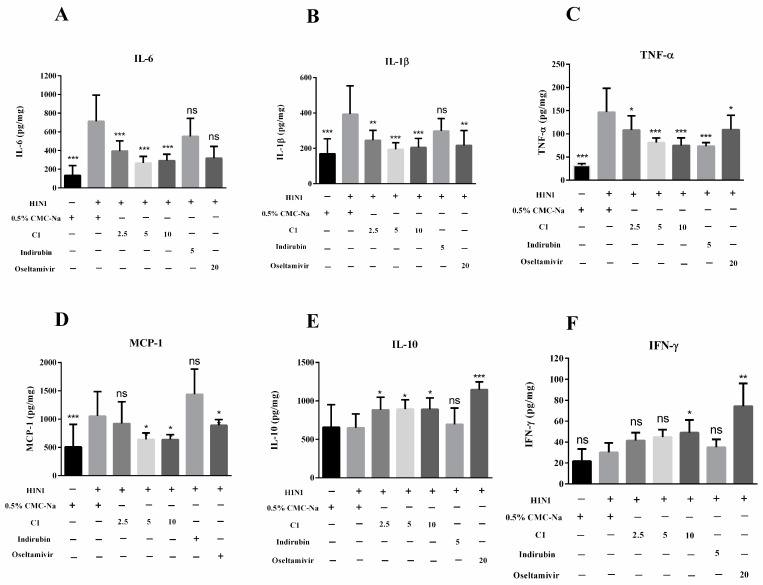
C1-alleviated lung injury in mice after 4 days of administration (*n* = 6, means ± S.D.); the experiment design is the same as Figure 3. Concentration of chemokines/cytokines/interferon was measured on day 4 post-infection by ELISA in lung homogenates (*n* = 6). (**A**) IL-6; (**B**) IL-1β; (**C**) TNF-α; (**D**) MCP-1; (**E**) IL-10; (**F**) IFN-γ. These data are representative of three experiments and are presented as the mean ± S.D. * *p* < 0.05, ** *p* < 0.01, *** *p* < 0.001, compared with the model group.

Alveolar macrophages located in pulmonary interstitium and alveolar cavity were considered components of the initial response to virus invasion and essential factors in the early clearance of pathogens and antigens. Subsequently, cytokines or chemokines were released, followed by increased neutrophil recruitment to disrupt or engulf the virus in the lung. The maintenance of cytokines at appropriate levels was helpful for controlling viral replication. However, at excessive levels, inflammatory cytokines induced lung immune injury and severe complications, contributing to the pathological course of viral pneumonia. Macrophages activated by IAV released inflammatory and anti-inflammatory cytokines, such as IL-1, IL-6, TNF-α and interferons [24]. Therefore, we evaluated their anti-inflammatory activity in C1-treated mice and quantified these cytokines in lung homogenates. In our study, cytokines were elevated in lung tissue homogenates from mice infected with fatal H1N1 virus. The results showed that the concentration of IL-6, IL-1β, TNF-α and MCP-1 (133.80 ± 61.30, 170.00 ± 48.38, 31.71 ± 2.44, 508.20 ± 180.30 pg/mg) was significantly higher after H1N1 attack, compared with the normal control (712.68 ± 99.30, 393.10 ± 60.70, 146.80 ± 16.24, 1054.00 ± 152.30 pg/mg) (*p* < 0.001). Compared with model group, treatment with C1 (2.5, 5, 10 mg/kg) and oseltamivir phosphate (20 mg/kg) significantly reduced the levels of IL-6, IL-1β and TNF-α and MCP-1 production in a dose-dependent manner (*p* < 0.001) (Figure 5). In addition, the expression level of IL-10 and INF-γ in the H1N1 virus group was significantly lower than in the normal mice (*p* < 0.05) but higher in mice treated with C1 (10 mg/kg) and Os. (20 mg/kg). We detected cytokines, chemokines and interferon in lung homogenates of mice at day 4 post-infection, among them, pro-inflammatory factors exhibited the most significant change after treatment with C1 and Os. As a common ingredient in indigo naturalis, indirubin was used as the control for comparing to the analogue of C1. Six cytokines in the indirubin group (5 mg/kg) were similar to those in the model control, which suggested that indirubin, unlike C1, could not block the excessive immune response induced by H1N1 infection.

**Figure 6:**
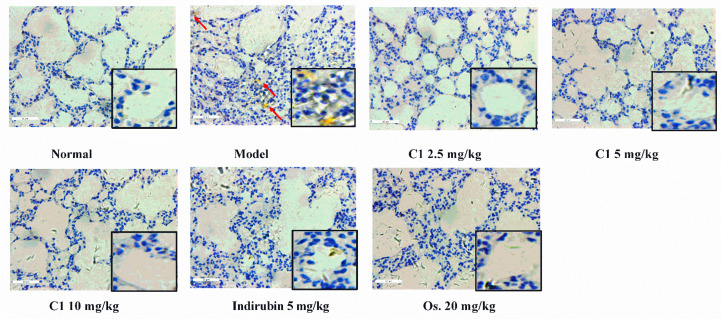
The effect of C1 on the recruitment of Iba-1 macrophages and monocytes in the ALI model; the experiment design is the same as Figure 3. The lung tissue of H1N1-infected mice was harvested, fixed in formalin, and embedded in paraffin and processed for slices preparation. The slides were incubated with relative antibodies against ionized calcium-binding adaptor molecule-1 (Iba-1), stained with a chromogenic substrate and counterstained with hematoxylin. The immuno-histochemical (×400) expression of Iba-1 in the lung of H1N1-infected mice was observed under a microscope. The scale bar represents 50 μm. Magnification: 400×; scale bars, 50 μm.

To confirm the relationship between inflammatory cytokines and macrophages, we evaluated the recruitment of macrophage using the IHC method. Inflammation is a complex process that is mediated by microglia and macrophage, which express ionized calcium binding adaptor molecule-1 (Iba-1) that produce the cytokines, including TNF-α and IL-1β [25]. As shown in Figure 6, microscopic evaluation revealed no obvious changes in the lungs from the control mice. In contrast, lungs from infected mice showed viral pneumonia characterized by deposition (solid arrows). Notably, fewer positive signals were observed in the lung tissues after C1 and indirubin administration. Taken together, these results demonstrate that C1 could inhibit the production of various inflammation cytokines and reduce the recruitment of alveolar macrophages in H1N1-infected mice, which exert anti-inflammatory activity.

## 4. Discussion

Influenza is an acute respiratory disease that poses a major health threat to the population. Therefore, there is an urgent need for a stable and effective treatment [26]. Traditional Chinese medicine is broadly used in the treatment of pyrexia, defervescence, inflammatory diseases and respiratory diseases, including pneumonia [27]. Our previous studies revealed that formulation QM decoctions with anti-oxidant and anti-inflammatory activity in vivo can attenuate the pulmonary injury induced by H1N influenza. To identify key active substances of formulation QM decoctions treating the IAV-infected mice, the bioactivity-oriented separation has been used to screen the active component from QM [28].

Our findings have shown that the EA extract from the formulation QM effectively attenuates H1N1-induced ALI in mice. In the H1N1 virus infecting the mice intranasally, EA extract (2.5, 5, 10 mg/kg) significantly reduced the lung index and prevented pneumonia damage in a dose-dependent manner. Moreover, EA extract inhibits the overproduction of inflammatory cytokines, including IL-6, TNF-α and MCP-1, in mice induced by viral pneumonia (Figures S1 and S2). Excessive immune reaction causing a cytokine storm is a harbinger of influenza-mediated death and triggers pathological injuries in lung tissues [29]. These observations suggest that potential components in EA extracts exert a significant therapeutic effect on inflammation and immune dysfunction induced by H1N1 in a mouse model. Furthermore, we analyzed the chemical constituents from the EA extract, which confirmed the major active compositions. The compounds identified above were tested for anti-virus in vitro. Specifically, LC-MS profiling revealed substantial alkaloids, monoterpenoids and nucleosides. As known compounds C1 and C3 were identified as alkaloids in indigo naturalis for the first time, C2 was identified as a monoterpenoid in indigo naturalis for the first time; C4 was identified as an alkaloid in Mabo. Due to the efficacy of EA extract in mitigating viral pneumonia in mice, we hypothesize that compounds from EA extract may exhibit identical biological activities in vitro. We performed an anti-virus test on MDCK cells to understand the antiviral activity of major components. Among these compounds, C1 exhibited excellent antiviral efficacy; the IC_50_ was 23.8 μg/mL; the IC_50_ of compound 3 against H1N1 was 100 μg/mL, the IC_50_ of ribavirin against H1N1 was 37.2 μg/mL. Meanwhile, the other compounds demonstrated weak activity. In vitro experiments confirmed that two compounds, and, specifically C1, exerted strong antiviral effects by suppressing viral replication, indicating their therapeutic capacity for ALI. In addition, its antiviral effects are rarely addressed, particularly in the treatment of viral pneumonia.

Studies suggest that indirubin is a prevalent bioactive ingredient in indigo naturalis with antiviral activity, and antitumor effects on various tumor cells [30]. Therefore, the indirubin group (5 mg/kg) was the control in this experiment. Additionally, oseltamivir (20 mg/kg) was used as a positive control instead of ribavirin (100 mg/kg), since the mechanism through which ribavirin acts as an antiviral is skeptical [31]. To verify our hypothesis, further investigations were performed in C1, indirubin and oseltamivir on mice infected by the H1N1 virus. As a consequence, C1 ameliorated lung edema, which was measured as lung index [32], whereas indirubin exerted minimal effect. Neuraminidase (NA) activity is an essential enzyme in the replication of the influenza H1N1 virus. Moreover, NA is responsible for releasing virions from infected cells. Nucleoprotein (NP) is an immunodominant antigen in numerous enveloped virus infections. In addition, nucleoproteins encapsulate viral RNA-forming ribonucleoprotein (RNP) particles in influenza A virus, which is critical in transcription, assembly, and packing [33]. Since NP and NA can reflect the ability of virus replication and transmissibility, we, therefore, evaluated the activity of NA and the expression level of NP in lung homogenates to evaluate its antiviral effect in vivo. At present, suppressing the role of neuraminidase is an effective therapeutic strategy for influenza. Based on our previous study, the viral load of infected mice was mitigated after treatment with C1; this was indicated by the down-regulated expression of neuraminidase and NP compared to that of model control. C1 effectively inhibits H1N1 replication in vitro and in vivo, as well as significantly alleviating lung injury. In addition, ELISA analysis revealed that C1 administration reduces various excessive inflammatory reactions in the host, as a result of H1N1 inhibition. Alveolar macrophages stimulated by influenza A produce inflammatory factors and mediators, which aggravate lung inflammation. C1 has a direct antiviral effect on influenza A, thereby restraining cytokine activation.

As expected, mice induced by H1N1 had a massive infiltration of inflammatory cells, including lymphocytes and neutrophils in the lungs and excessive cytokines released from inflammatory cells, both of which synergistically inhibit viral replication. Therefore, inhibiting the overproduction of the H1N1 virus may promote the blocking of subsequent inflammatory reactions. We, therefore, validate the aforementioned hypothesis.

We found that in mice treated with C1, influenza virus symptoms were mild, lung edema and white blood cell recruitment were mitigated, excessive cytokines levels in lung tissue were inhibited and the overall pathology was ameliorated, indicating an effective antiviral therapeutic approach for a future pandemic of the influenza A (H1N1) virus. Our findings also indicate that the anti-viral activity of C1 reduces the release of both pro-inflammatory cytokines and chemokines. As such, there is an urgent need to explore the mechanism by which highly pathogenic viruses cause disease. Studies have reported that ALI in H1N1-infected mice is induced by excessive inflammatory cell infiltration and activation, including the release of proinflammatory factors and chemokines. Of note, the influenza virus is the pathogen of influenza. We obtained the C1 using the bioactivity-oriented separation method, which is a therapeutic constituent of viral pneumonia under the animal models in vitro and in vivo. For the first time in Chinese herbal medicines, C1 was found in the indigo naturalis. C1 reduces the NA and NP expression of influenza A virus and excessive cytokine activation, which is promising in the prevention and treatment of influenza virus. Because of its quinazolinone structure, C1 is categorized as an alkaloid. Therefore, C1 might be a potential compound for a novel anti-flu drug; however, its antiviral mechanism warrants in-depth research.

## 5. Conclusions

For the first time, C1 was confirmed as a major component of the EA extract of QM to exert antiviral activities in vitro and in vivo. Our results revealed that the compound is responsible for the inhibition of H1N1 replication and effectively alleviates H1N1-induced ALI in mice, which was related with its antiviral properties. These results suggested that C1 is main active compound in the traditional Chinese medicine QM, which deepens the understanding of the pharmacodynamic material basis of QM. The results provide an alternative scheme for ALI therapy induced by the influenza A virus.

## Figures and Tables

**Table 1:** Anti-IVA of C1.

Compound	TC_50_ (μg/mL)	IC_50_ (μg/mL)
C1	79.7	23.8
C2	26.7	/
C3	106.8	100
C4	167.7	/
Ribavrin	/	37.2

## Data Availability

Data will be provided upon request.

## Supplementary Materials

The following supporting information can be downloaded at: https://www.mdpi.com/article/10.3390/molecules27227857/s1. Figure S1: Determination of virulence of H1N1 virus using the LD_50_ method; Figure S2: The protective effects of EA extract on H1N1virally infected ALI; Figure S3: Effects of EA extract on cytokines in pulmonary tissue homogenate of mice; Figure S4: UPLC−LTQ−MS total ion chromatogram of EA extract in positive-ion mode; Table S1: Ingredient compounds identified in EA extract from the QM water aqueous according to Molecular formula; M^1^; M^2^; t_R_. References [34,35,36,37,38,39] are cited in the Supplementary Materials.
