# Supplementary material for: Therapeutic Potential of 2-Methylquinazolin-4(3H)-one as an Antiviral Agent against Influenza A Virus-Induced Acute Lung Injury in Mice
Source: Molecules. 2022 Nov 14;27(22):7857. doi: 10.3390/molecules27227857 (PMC9697438; doi:10.3390/molecules27227857)
Supplement: Supplementary file 1 [file molecules-27-07857-s001.zip › molecules-1989061-supplementary materials.pdf]

Therapeutic potential of 2-Methylquinazolin-4(3H)-one as an antiviral agent against influenza A virus-induced acute lung injury in mice

Rong Tian [1], Haiyan Zhu [2,*], Yan Lu [1], Xunlong Shi [2], Peng Tu [1], Hong Li [3], Hai Huang [2] and Daofeng Chen [1,*]

[1] Department of Natural Medicine, School of Pharmacy, Fudan University, No. 3728, Jin Ke Road, Shanghai 201203, China
[2] Department of Biological Medicines & Shanghai Engineering Research Center of Immunotherapeutics, School of Pharmacy, Fudan University, No. 3728, Jin Ke Road, Shanghai 201203, China
[3] Department of Pharmacy, Fudan University, No. 3728, Jin Ke Road, Shanghai 201203, China
* Correspondence: haiyanzhu@fudan.edu.cn (H.Z.); dfchen@shmu.edu.cn (D.C.)

1. Supplementary methods

1.1. LD_{50} survival experiment

BABL/c mice were anesthetized with isoflurance using a gas anesthesia apparatus. To determine the LD_{50}, mice were randomly divided into 7 groups (n = 10 for each group) and intranasally challenged with IAV in 30 μL of RPMI-1640 medium as follows: for comparison, the normal group was given RPMI-1640 medium, the other H1N1 group inhaled with H1N1 virus at with 10^{-2}, 10^{-3}, 10^{-4}, 10^{-5}, 10^{-6} respectively. All groups were monitored for 14 days after viral infection. Body weight, temperature, and animal clinical heath were monitored daily. Moreover, we recorded the lifespan and mortality rates of mice. The Reed-Muench formula was used to measure the 50% lethal dose (LD_{50}) H1N1 influenza virus.

1.2. *In vivo* experiments of EA on ALI induced by H1N1 influenza virus

EA or Ribavirin (Rb) was dissolved in a 0.5% sodium carboxymethyl cellulose (CMC-Na) solution for *in vivo* experiments. EA was diluted to a final concentration of 2.5, 5, and 10 mg/kg. Rb was diluted to 100 mg/mL. EA (2.5, 5, and 10 mg/kg). Rb dissolved in CMC-Na was administered to mice once per day (i.g.) for 4 days after inhaling H1N1 ($3LD_{50}$). Meanwhile, normal and model control were administered with equivalent amounts of 0.5% CMC-Na solution once a day for 4 consecutive days. The mice were sacrificed after treatment with EA or Rb for four days. The lung lesions were dissected, and we measured the body and lung weights. Blood plasma and tissue samples were collected for subsequent experiments. The study protocol can be referred to as 2.6.1.

1.3. Effects of MQ on the levels of IL-6, TNF-α, MCP-1, and IL-10 in lung supernatant

The same method descriptions can be referenced in 2.6.3.

1.4. Lung index ratio and hematoxylin and eosin (H＆E) staining

The same method descriptions can be referenced in 2.6.1.

1.5. Characteristics of chemical constituents in EA extract

The same method descriptions can be referenced in 2.2

2. Supplementary results

2.1. Lethal dose, 50% (LD_{50})

The mice were subjected to the lethality of the H1N1 virus at 10^{-2}, 10^{-3}, 10^{-4}, 10^{-5}, and 10^{-6} then observed for 14 consecutive days. Among the various groups, the experimental results revealed that mice infected with the H1N1 pathogens had decreased body weight and higher mortality rates (Figure S1). Specifically, the 10^{-2} H1N1 group achieved the highest mortality rate (90%), indicating that H1N1 infection for 4 days caused severe sickness. The LD_{50} was $10^{-5.56}$ mg/kg which was obtained using the Reed-Muench formula. Our findings provide a reasonable parameter of the live mouse-adapted H1N1 virus (3LD_{50}) for subsequent research.

2.2. Beneficial effects of EA extract on influenza H1N1-induced ALI

The total EA extract content of QM decoctions was 5% (EA extract, g / the MQ decoctions, g). The mice were infected with 3 × LD_{50} of the H1N1 virus and then treated with 2.5 mg/kg, 5 mg/kg, 10 mg/kg of body weight/day of EA or 100 mg/kg Ribavirbin as a positive control once daily via intragastric administration (i.g) for 4 days. The lung index revealed the severity of lung lesions; therefore, lung weight and lung index were recorded and measured to establish the effects of EA extract on suppressing lung edema. As shown in Figure S2A, significant increase was observed in lung index model control (11.58 ± 0.39 mg/g) compared with that of normal control (6.28 ± 0.39 mg/g), $p < 0.001$. Most strikingly, unlike the model group, treatment with EA extract 2.5, 5, 10 mg/kg and Ribavirbin 100 mg/kg attenuated edema and exudation in the lungs (9.68 ± 0.68, 9.43 ± 0.28, 8.70 ± 0.28 and 7.32 ± 0.39 mg/g, respectively), $p < 0.001$. The results confirm that EA extract ameliorates lung edema and severity of lung lesion in virus-infected mice from 2.5 to 10 mg/kg in a dose-dependent manner, hence indicating more critical components in viral clearance from infected lungs. As is shown in Figure S2B, liver index of mice in model group (50.73 ± 0.58 mg/g) was significantly lower than that in the normal control (62.79 ± 1.24 mg/g), $p < 0.05$; EA extract (5, 10 mg/kg) and Ribavirbin (100 mg/kg) increased the weight of liver index (52.09 ± 1.15, 52.02 ± 1.20 and 59.60 ± 1.68 mg/g, respectively), whereas 2.5 mg/kg dose did not increase the liver weight. In our research, severe injury of vital organs, including lung, liver were recorded in IAV-induced mice. The results show that administration of EA extract attenuated injury of vital organs, including increase liver weight. Besides, body weight was observed and recorded for 5 days (Figure S2C). The normal group and Ribavirbin control gained weight from day 1 to day 5, whereas, the mice-infected virus lost weight from day 2 after inoculation. The EA extract (10 mg/kg) administration group had a mild appetite and weight loss. All the infected mice revealed a sign of disease symptoms, including weight loss, dull fur, inactivity, and respiratory distress.

2.3. Effect of EA extract on cytokine production and lung tissue histological changes of mice infected with H1N1

The pro-inflammatory cytokines TNF-α, and IL-6 are the most promising biomarkers for morbidity and mortality prediction. The level of 4 cytokines was determined in the lung supernatant (Figure S3A). The levels of IL-6, TNF-α, and MCP-1 dramatically increased in lung homogenates than that in normal control ($p < 0.001$). Treatments with EA extract (2.5, 5 mg/kg) significantly reduced their overproduction compared with the model group, whereas administration with 10 mg/kg decreased the pro-inflammatory cytokine, with a statistically insignificant difference (IL-6, TNF-α). Meanwhile, IL-10 levels dramatically increased after administering EA extract, compared

with the model group ($p < 0.001$). The aforementioned data indicate that EA extract inhibits the inflammatory response in the lung and attenuates pathology damages.

H & E staining was performed on lung tissues to evaluate the histological changes in H1N1 virus-infected mice after administration with EA extract. As shown in supplementary data Figure S3B, microscopic analysis revealed that lungs from the normal group had no remarkable changes in epithelial cells and pulmonary alveoli. In contrast, the lung tissues from the model group revealed widespread alveolus damage, thickened alveolar, interstitial edema, neutrophil and lymphocyte infiltrate in the alveoli. The lung tissues of EA extract and Ribavirin group presented relieved tissue consolidation with a slight hemorrhage, and a small amount of interstitial lymphocyte infiltration compared to the infected group.

2.4. Qualitative analysis of material in the EA extract

2.4.1. Identification of alkaloids

Among the chemical structure (Figure S4), alkaloids are the primary type of chemical compound in EA extract, and potentially the active constituents which play a vital role in the treatment of virus-ALI. The study [34, 35] shows that this phytochemical component is active *in vitro* against the HIV, TMV, and HUH-7 cancer cell lines. Based on the reference of quinazolinone derivatives, protonated molecular ion of this compound lose 28 or 16 Da [36]. Herein, most alkaloids have similar MS fragmentation behaviors, such as peaks (1, 2, 4, 5, 6, 10-18,). These compounds produced characteristic neutral common fragment 28 Da which generated by quinazolinone derivatives (Table S1). For instance, compound 2, at *m/z* 133.12 derives from at *m/z* 161.19 lost CO, exhibited in the positive-ion spectra then lost CH_3 to generate a fragment at *m/z* 118.15, or lost C_2H_3N to appear the daughter ion at *m/z* 120.20.

2.4.2. Identification of nucleosides

We detected 1 nucleoside which was linked to adenine. Generally, this compound appears to MS responses in positive ion mode, compound 9 yielded $[M+H]^+$ at *m/z* 136.08 in positive ion mode, then lost NH_3 to generate the fragment at *m/z* 119.14, which is consistent with ion fragment and literature [37].

2.4.3. Identification of monoterpenes

Further, 2 monoterpenes were discovered in positive ion mode; compounds 3 and 7 were Annuionone D and Loliolide, respectively. Compound 3 formed $[M+H]^+$ at *m/z* 197.25 in positive ion mode, and its protonated molecular ion was broken into two parts by MS^2. Therefore, we detected the daughter ions at *m/z* 179.2 and *m/z* 135.17. Based on the above information, compound 3 can lose H_2O and CO_2 of Loliolide. Furthermore, Lolilide is a potent inhibitor of the hepatitis C virus [38]. Similarly, compound 7 was also characterized at the same time.

2.4.4. Identification of sterols

According to the reference to steroids [39], protonated molecular ions of this constituent can produce 112 Da. In this work, unknown steroids (8) have similar MS fragmentation behaviors, compound 8 at *m/z* 337.25 produced the ion 225.12 and lose 112 Da using the ESI-MS, which may be generated by steroid groups.

Table. S1 Ingredient compounds identified in EA extract from the QM water aqueous according to Molecular formula; M^1; M^2; t_R.

**Peak No.**	**Identification (possible chemical)**	**Categories**	**Formula**	**MS¹**	**t_R (min)**	**MS²**
1	2-Methylquinazolin-4(3H)-one	Alkaloids	$C_9H_8N_2O$	161.19	16.47	133.1, 118.14,
2	Loliolide	Monoterpenes	$C_{11}H_{16}O_3$	197.25	22.52	179.21, 135.17
3	3-(2-Hydroxyphenyl)-2-methyl-4(3H)quinazolinone	Alkaloids	$C_{15}H_{12}N_2O_2$	253.27	29.47	120.14, 134.11
4	2(1H)-Quinolinone,4-methoxy-1-methyl-3-(3-methyl-2-buten-1-yl)-	Alkaloids	$C_{16}H_{19}NO_2$	258.15	38.72	240.10
5	3H-Quinazolin-4-one	Alkaloids	$C_8H_6N_2O$	147.21	14.59	130.09
6	Quinazolinone	Quinazolinone alkaoids	—	299.33	33.09	271.25
7	Annuionone D	Monoterpenes	$C_{13}H_{20}O_3$	225.31	36.21	156.35
8	Sterol	Sterols	$C_{20}H_{32}O_4$	337.25	47.11	319.34, 225.12
9	Adenine	Nucleosides	$C_5H_5N_5$	136.15	5.45	119.14
10	Alkaloids	Alkaloids	$C_6H_7N_5$	150.07	9.11	108.04
11	Alkaloids	Alkaloids	C_9H_7NO	146.06	11.82	118.06
12	Quinazolinone	Quinazolinone alkaoids	$C_{10}H_9NO$	160.07	15.19	117.35
13	2H-Benzimidazol-2-one,1,3-dihydro-	Alkaloids	$C_7H_6N_2O$	135.05	19.81	107.05
14	Ethanone,1-(3-methyl-2-quinoxalinyl)-	Alkaloids	$C_{11}H_{10}N_2O$	187.08	20.69	145.23
15	Isaindigotone 2	Alkaloids	$C_{20}H_{18}N_2O_4$	351.42	41.57	333.36, 315.36
16	Cyclo	Alkaloids	$C_{11}H_{18}N_2O_2$	211.26	17.93	194.11, 183.22
17	1H-indol-3-yl) oxoacetamide	Alkaloids	$C_{10}H_8N_2O_2$	189.23	25.23	172.12, 168.33
18	Tymine	Alkaloids	$C_5H_8N_2O_2$	129.63	2.53	129.6, 117.10

Identification of the chemical constituents of EA extract by UPLC-ESI-MS^n in positive-ion mode and part of identification were confirm with standards (1, 2, 3, 5, 15, 16)

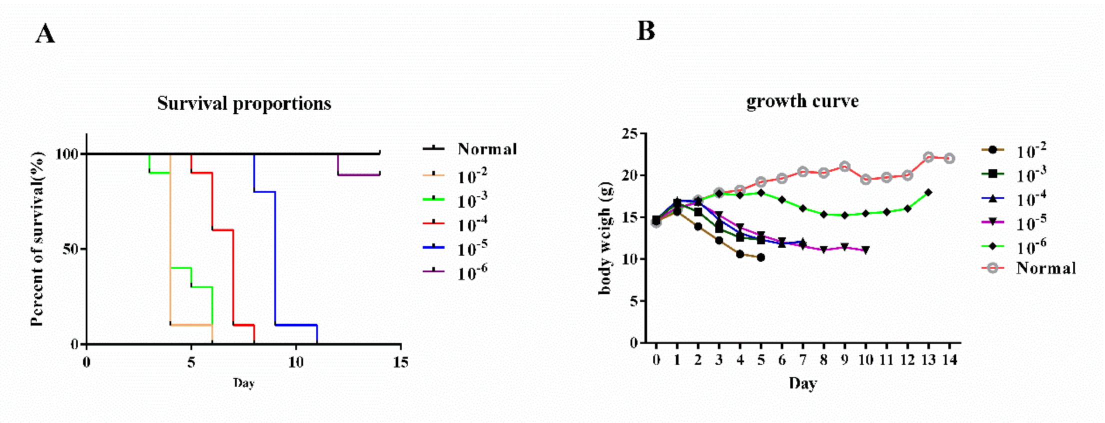

Figure.S1 Determination of virulence of H1N1 virus using the LD_{50} method.

(A) Survival rate of various concentration groups; (B) Body weight change during 14 observing days. The mouse-adapted virus was obtained using 4 serial passages in the lungs of BALB/c mice; thereafter, 10 mice in each group were infected intranasally with H1N1 virus at with 10^{-2}, 10^{-3}, 10^{-4}, 10^{-5}, 10^{-6} and were monitored for 14 days for a sign of morbidity (body weight changes, hunched posture, and fever) and mortality, untreated mice were used as an additional negative control. The LD_{50} was calculated using the Reed-Muench formula, and the LD_{50} was $10^{-5.56}$ mg/kg; then, mice were challenged with a 3 × LD_{50} dose of the live mouse-adapted H1N1 virus in the following study.

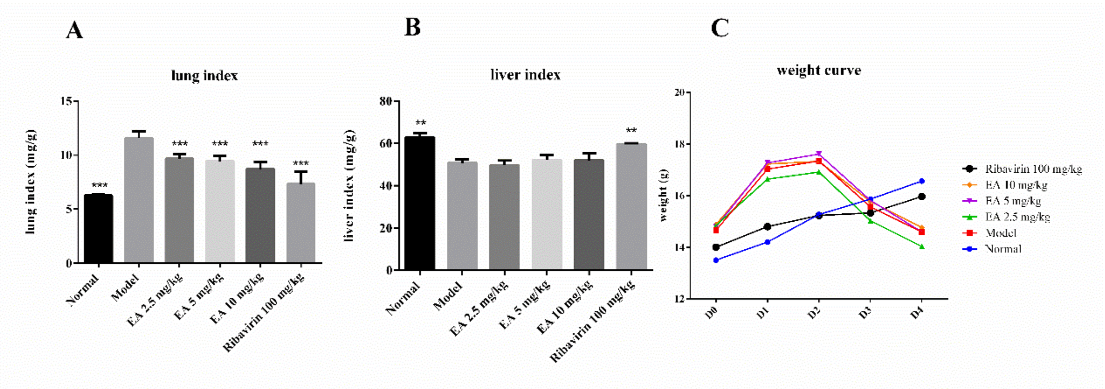

Figure.S2 The protective effects of EA extract on H1N1virally infected ALI. Mice were infected with 3 × LD_{50} and administered orally with EA extract, ribavirin, or 0.5% CMC-Na at indicated doses once daily for 4 days. Lung index and mice body weight were measured and calculated. (A) Lung index, (B) Weight of liver, (C) Body weight of 5 days. Data are presented as Mean ± S.D. * $P < 0.05$, ** $P < 0.01$, *** $P < 0.001$ compared with the virus model group.

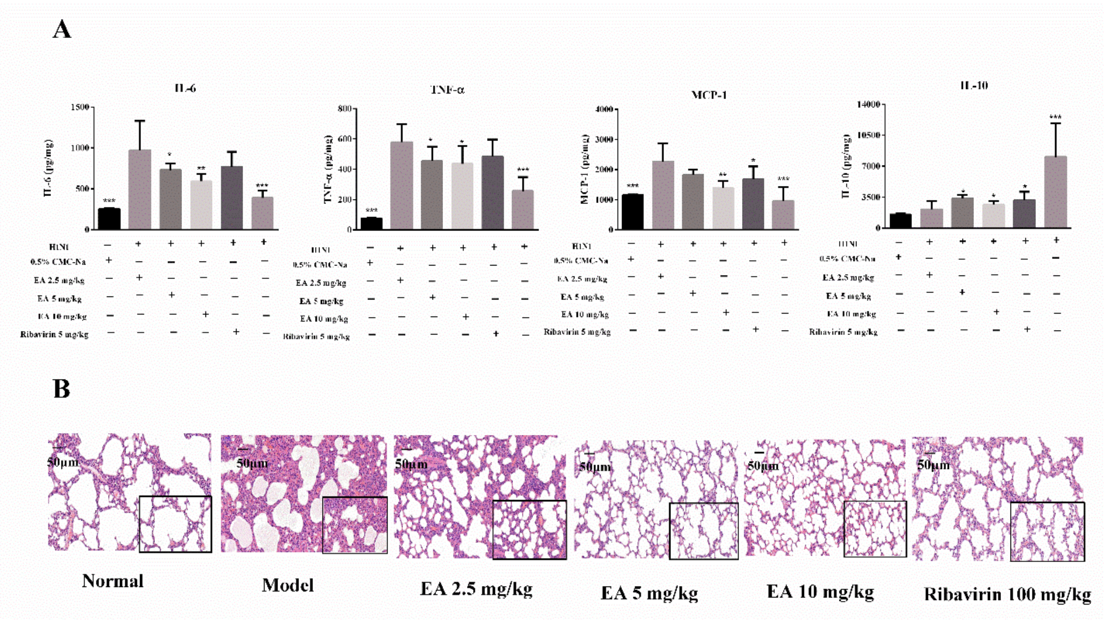

Figure.S3 Effects of EA extract on cytokines in pulmonary tissue homogenate of mice.

(A) IL-6; (B) TNF-α; (C) MCP-1; (D) IL-10; Data were presented as Means ± S.D. (n=6). (E) Hematoxylin-eosin stained lung tissues sections in each group with 200 ×, 400 × magnification (Plotting scale = 50 μm)

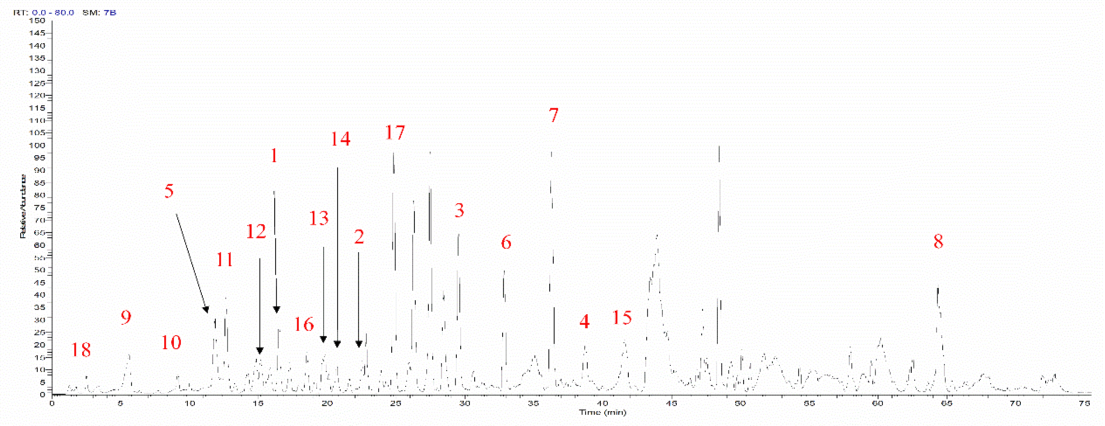

Figure.S4. UPLC−LTQ−MS total ion chromatogram of EA extract in positive-ion mode. EA extract was diluted in methanol and then filter through a 0.22 μm filter membrane before being introduced to the UPLC-ESI-LTQ-MS analysis. A YMC-Triart C_{18} column was used with an injection volume of 5 μL for the components separation and analysis in positive ion mode.

34 Gao, X.; Cai, X.; Yan, K.; Song, B.; Gao, L.; Chen, Z. Synthesis and antiviral bioactivities of 2-aryl- or 2-methyl-3-(substituted-benzalamino)-4(3H)-quinazolinone derivatives. *Molecules* **2007**, *12*, 2621–2642.

35 Akgun, H.; Us Yilmaz, D.; Cetin Atalay, R.; Gozen, D. A Series of 2,4(1H,3H)-Quinazolinedione Derivatives: Synthesis and Biological Evaluation as Potential Anticancer Agents. *Lett. Drug Des. Discov.* **2016**, *13*, 64–76.

36 Smith, K.; El-Hiti, G.A.; Abdel-Megeed, M.F. Unexpected Products from Carbonylation of Lithiated Quinazolin-4(3H)-one Derivatives. *Russ. J. Org. Chem.* **2003**, *34*, 430–435.

37 Van Dycke, A.; Verstraete, A.; Pil, K.; Raedt, R.; Vonck, K.; Boison, D.; Boon, P. Quantitative analysis of adenosine using liquid chromatography/atmospheric pressure chemical ionization-tandem mass spectrometry (LC/APCI-MS/MS). *J Chromatogr. B* **2010**, *878*, 1493–1498.

38 Chung, C.-Y.; Liu, C.-H.; Burnouf, T.; Wang, G.-H.; Chang, S.-P.; Jassey, A.; Tai, C.-J.; Tai, C.-J.; Huang, C.-J.; Richardson, C.D.; et al. Activity-based and fraction-guided analysis of Phyllanthus urinaria identifies loliolide as a potent inhibitor of hepatitis C virus entry. *Antivir Res.* **2016**, *130*, 58–68.
39 Zhao, Y.Y. The Study of Chemical Constituents and Bioactive Constituents of *Polyporus umbellatus*. Ph.D. Thesis, Northwest University, Xi'an, China, 2010.
